# High-Throughput GLP-Capable Target Cell Visualization Assay for Measuring Cell-Mediated Cytotoxicity

**DOI:** 10.3390/cells7050035

**Published:** 2018-04-24

**Authors:** Anna Welter, Srividya Sundararaman, Ruliang Li, Ting Zhang, Alexey Y. Karulin, Alexander Lehmann, Villian Naeem, Diana R. Roen, Stefanie Kuerten, Paul V. Lehmann

**Affiliations:** 1 Research & Development Department, Cellular Technology Limited, Shaker Heights, OH 44122, USA; anna.welter@web.de (A.W.); svidya.sraman@gmail.com (S.S.); ruliang.li@immunospot.com (R.L.); ting.zhang@immunospot.com (T.Z.); alexey.karulin@immunospot.com (A.Y.K.); alexander.lehmann@immunospot.com (A.L.); villian.naeem@immunospot.com (V.N.); diana.roen@immunospot.com (D.R.R.); 2 Institute of Anatomy and Cell Biology, Friedrich-Alexander University Erlangen-Nürnberg, 91054 Erlangen, Germany; stefanie.kuerten@fau.de

**Keywords:** TVA, NK measurement, ADCC, CD8 cells, multi-color single cell imaging, CFR-Part 11 compliance, audit trails, cellular assay validation, immune monitoring

## Abstract

One of the primary effector functions of immune cells is the killing of virus-infected or malignant cells in the body. Natural killer (NK) and CD8 effector T cells are specialized for this function. The gold standard for measuring such cell-mediated cytolysis has been the chromium release assay, in which the leakage of the radioactive isotope from damaged target cells is being detected. Flow cytometry-based single cell analysis of target cells has recently been established as a non-radioactive alternative. Here we introduce a target cell visualization assay (TVA) that applies similar target cell staining approaches as used in flow cytometry but based on single cell computer image analysis. Two versions of TVA are described here. In one, the decrease in numbers of calcein-stained, i.e., viable, target cells is assessed. In the other, the CFSE/PI TVA, the increase in numbers of dead target cells is established in addition. TVA assays are shown to operate with the same sensitivity as standard chromium release assays, and, leaving data audit trails in form of scanned (raw), analyzed, and quality-controlled images, thus meeting requirements for measuring cell-mediated cytolysis in a regulated environment.

## 1. Introduction

For the past few decades, immune monitoring has primarily served basic scientific research, with the goal of understanding of the immune system’s workings. In recent years, however, advances in the treatment of human diseases, foremost of these being cancer, with monoclonal antibodies, adoptive cell therapies, vaccinations and Chimeric Antigen Receptor transfected T cells (CAR-T), have not only revolutionized medicine but have also introduced new requirements for immune monitoring [1]. Unlike in academic research, where there are limited regulatory requirements, regulated immune monitoring trials that support the development of treatment protocols or therapeutic decisions require the provision of tamper-proof audit trails for the data generated [2].

Among the methods that permit the generation of immune monitoring data suitable for work in a regulated environment, ELISPOT was among the first to meet this requirement [3]. The reasons are a unique combination of (a) the high sensitivity of this assay for detecting low-frequency antigen-specific T cells; (b) its adaptability to measure various T-cell derived cytokines including their co-expression patterns; (c) the reproducibility of the assay; (d) its scalability for high-throughput, and most of all; (e) the transparency of the data and its analysis. ELISPOT assay plates can be scanned and counted in a tamper-proof fashion documenting the assay results. The actual analysis can be done in an automated yet accurate fashion, while automatically recording each step of the analysis, and the eventual quality control step is performed with insuppressible annotations keeping audit trails for traceability of the final results [4]. In this publication, we have set out to adopt the very same platform for high-throughput capable measurements of cell-mediated cytotoxicity, while automating the analysis process and keeping, as regulations require, 21 CFR Part 11 conforming audit trails of every step of it using the very instrumentation used for ELISPOT analysis.

Cell-mediated cytolysis by Natural Killer (NK) cells and CD8+ T cells, as well as antibody dependent cell-mediated cytotoxicity (ADCC), has been traditionally measured by the so-called Chromium (^51^Cr)-Release Assay, CRA [5], which remains the gold standard read-out system.

In addition to the use of a radioactive isotope, ^51^Cr, increasingly under regulatory scrutiny for reasons of workplace and environmental safety, the CRA has several other shortcomings; foremost for its relevance in immune monitoring trials that it does not allow for the retention of fully transparent audit trails for regulated trials.

Another shortcoming of the CRA is that it is semi-quantitative: instead of enumerating live and/or dead target cells directly, only the amount of ^51^Cr released by dead or dying target cells is recorded as a percentage of that released by total (control) target cell lysis. Herein lays an inherent weakness of semi-quantitative assays that is illustrated in Figure S1. In cytolytic assays, a fixed number of labeled target cells are admixed with effector cells, titrating the number of the latter. The effector cells are typically peripheral blood mononuclear cells (PBMC), among which the actual effector cells are rare. When measuring NK activity, NK cells constitute typically a couple percent of cells within PBMC—antigen-specific CD8 cells occur in frequencies orders of magnitudes lower [6]. In cytolytic assays, these rare effector cells among PBMC need to seek out and kill target cells. Thus, contact between effector and target cells is a rate-limiting step. Additionally, in semi-quantitative assays such as the CRA, by convention more than 20–30% of target cells need to be killed for the result to be accepted as positive [5]. For example, if a typical number of 5 × 10^3^ target cells are plated per well, at least 1 × 10^3^ of these need to be contacted and killed by the rare effector cells before killing becomes reliably detectable. Therefore, assays that detect killing at the single cell level are preferable.

A number of assays have been developed aiming at measurements of cytolytic activity at the single-cell level. One variant of these assays measures, in ELISPOT format, the release of granzyme B [7] or perforin [8] by effector cells that engage in cytolysis of their target cells. During the exocytosis of these effector molecules, the cytolytic granule constituent CD107 is transitioned to the surface of the effector cells, and thus can be detected by flow cytometry [9]. Other single-cell variants of cytotoxicity assays measure, also by flow cytometry, the percentage of fluorescence-marked target cells that survive exposure to the effector cells, e.g., of GFP transfected target cells [10], or of targets labeled with dyes that are released as the cell dies [11]. Perhaps the most intriguing approach to detecting cell-mediated cytolysis in vitro is based on the detection of target cells that have been killed: if this method is sensitive enough, it should no longer be a requirement that a high number (20–30%) of the target cells are killed before cytolytic activity becomes unambiguously detectable. In this case, even relatively few killed target cells could provide clearly quantitative evidence for ongoing cytolytic activity. The use of fluorogenic caspase substrates, for example, permits the visualization of targets cells undergoing apoptosis [12].

While such flow cytometric measurements of killing activity have represented a major step towards the advancement of immune monitoring, the use of the flow cytometry has inherent drawbacks. First, flow cytometry is challenging when it comes to target cells that are adherent to plastic. Second, while data acquisition can be automated (at substantial cost), the data analysis itself remains manual and time-consuming, requiring highly trained personnel making subjective judgment calls relating to compensation and setting of gates. Based on fluidics, the cost and effort of flow cytometer calibration and maintenance are substantial. Also the efficiency of cell utilization is suboptimal in flow cytometry, as cells get lost in the tubing of the instrument.

In the present study, we have set out to demonstrate the feasibility of single-cell analysis for measurements of cytolytic activity, i.e., the enumeration of live and dead target cells using image cytometry. In this approach, fluorescent dye-labeled target cells are imaged within the wells of a 96-well plate, the image of the well containing the labeled cells is captured in a tamper-proof format, thus keeping an audit trail for the raw data, then fully automated image analysis follow recognizing and counting live and dead target cells. Also during this analysis process full tamper-proof audit trails are generated automatically documenting the accuracy of the object recognition (i.e., live/dead cell-target cell counting), and the results are automatically plotted as graphs showing the percentage of killing at various effector/target (E:T) ratios. An optional quality control (QC) step is supported permitting the operators to view and revise the results of the automated counting process, however, again automatically leaving an insuppressible audit trail with annotations as to the changes made, thus meeting regulatory requirements. Using this platform, it takes about one minute of fully automated reader time to come up with the test results for a PBMC sample: scanning and counting the cells in 24 wells of a 96 well plate (with three replicate measurements for each E:T ratio), documenting the data and printing publication-ready graphs (an example of which is shown, e.g., in Supplementary Figure S2A), while audit trails are automatically established for each step of the process. Thus, TVA assays are scalable for high-throughput testing in a regulated environment.

In particular, we focused on two types of readout for cytotoxicity. One is based on calcein staining of target cells. As live target cells retain calcein, while dying and dead cells leak the dye, one can directly count how many live target cells are present in a given well at the end of the assay and, knowing how many target cells were plated at the beginning of the assay, one can calculate % killing. In the other approach, we use carboxyfluorescein succinimidyl ester (CFSE, green) and propidium iodide (PI, red) fluorescence staining to count live target cells (CSFE single positive) and dead target cells (CFSE + PI double positive).

## 2. Materials and Methods

### 2.1. PBMC Donors

Cryopreserved PBMC from 40 healthy donors (age 22–45) were selected based on NK activity pre-screening criteria from the ePBMC library (CTL, Shaker Heights, OH, USA: www.immunospot.com/ImmunoSpot-ePBMC). These donors were recruited by Hemacare (Van Nyus, CA, USA) and their PBMC isolated by leukapheresis using Hemacare IRBs. The PBMC were then cryopreserved at CTL and stored in vapor liquid nitrogen until testing [13]. Thawing, washing, and counting of the cryopreserved cells was done according to an optimized protocol [14]. After thawing, the viability of PBMC, as established by the CTL LD (live/dead cell) counting platform of the ImmunoSpot^®^ S6 FluoroCore Reader (CTL) was invariably greater than 85%. Within 4 h after thawing, the cells were plated for the TVA assays. In select experiments, the NK cell subset was depleted from PBMC using a magnetic bead selection kit (Stem Cell Technologies, Vancouver, BC, Canada). The depletion assay was performed according to manufacturer’s instructions.

### 2.2. Antigens

As a positive control for PBMC functionality after NK cell depletion we tested for CD8 cell functionality using the CEF peptide pool that consists of 32 HLA Class I molecule—restricted peptides [15]. This peptide pool, widely used as a positive control for CD8 cell activation, was from CTL (CEFpp+, Cat. # CTL-CEF-002). The CEF peptide pool was added to the ELISPOT test system at a final concentration of 0.25 µg/mL.

### 2.3. Human IFN-γ-ELISPOT Assays

The functionality of the freeze-thawed PBMC and of the NK-depleted PBMC was verified by performing human interferon-γ ELISPOT assays using the ImmunoSpot^®^ Test Kit (CTL, Cat # hIFNG-1M) as described in [16]. In brief, the PVDF membrane was pre-coated with the anti-IFN-γ capture antibody overnight. CEF antigen was plated in 100 µL of CTL-Test Medium^TM^ (CTL, Cat #CTLT 005) while negative control wells contained this medium alone plus PBMC. The plates containing the antigen were stored in an incubator until the PBMC were ready for plating. The thawed PBMC were added in CTL-Test Medium^TM^ at 4 × 10^5^/cells per well in 100 µL. The plates were incubated for 24 h at 37 °C in a humidified incubator at 9% CO_2_. After discarding the PBMC, anti-IFN-γ detection antibody was added and the membrane-bound IFN-γ was visualized by an enzymatic reaction. The plates were dried prior to analysis. The ELISPOT plates were analyzed using an ImmunoSpot^®^ S6 FluoroCore Reader from CTL.

### 2.4. Target Cell Line

Target cells were K562 cells, a human chronic myeloid leukemia cell line (from ATCC). K562 cells were cultured in RPMI 1640 medium (Invitrogen, Carlsbad, CA, USA) supplemented with 10% FBS (Life Technologies, Carlsbad, CA, USA), and 1% Penn/Strep plus 1% l-glutamine (Life Technologies), at 37 °C in a 5% CO_2_ incubator.

### 2.5. The 96 Well Plate Calcein TVA Assay

#### 2.5.1. Calcein Staining of Target Cells

K562 cells were stained with an NK-D dye (CTL), a ready to use calcein-containing solution that has been optimized for imaging. Cells were stained by adding 1 µL of NK-D dye to 1 × 10^6^ K562 cell resuspended in 1 mL of CTL-Test Medium, and were incubated for 10 min in a 37 °C incubator, at 5% CO_2_. Following incubation, 5 mL of CTL-Test Medium was added to the cell suspension and the cells were centrifuged at 1200 RPM for 10 min. The supernatant was discarded and the cell pellet was tapped to re-suspend. The cells were washed two more times with CTL-Test Medium after which the K562 cells were counted using the ImmunoSpot S6 FluoroCore Reader (CTL) using the CTL L/D Cell Counting software suite to determine the number of stained live cells in the sample. The labeled target cells were then resuspended at 5 × 10^4^ cells/mL in CTL-Test Medium in order to plate them at 5 × 10^3^ cells/well (or the cell numbers specified), in 100 µL per well in a U bottom 96 well plate.

#### 2.5.2. The Cytotoxicity Assay

Effector cells (cryopreserved PBMC), were plated at 5 × 10^5^ cells/well in 100 µL of CTL-Test Medium into the first wells of a U bottom 96 well microtiter plate (resulting in the 100:1 E:T ratio), and serially diluted in the subsequent 6 wells of a column. The last well in the column had no effector cells present but contained CTL-Test Medium alone, serving as the negative control. The stained target cells were added at 5 × 10^3^ cells/well (or as specified) in 100 µL of CTL Test medium to all wells. The plate was centrifuged at 1200 RPM for 5 min and then incubated at 37 °C in a 5% CO_2_ incubator for 4 h. After the 4 h incubation, cells were gently resuspended in the wells and 50 µL of the cell suspension was transferred to a flat bottom 96-well imaging plate (available with the NK Kit, CTL), in triplicates. The imaging plate was analyzed by the NK-TVA Software Suite of the S6 FluoroCore Analyzer. This software automatically generates the killing curves based on the number of live cells in each experimental well, comparing it to the number of target cells in the negative control wells (no effectors).

#### 2.5.3. The Terasaki Plate Calcein TVA Assay

The target and effector cells were prepared as described as above for the 96 well assay, but only one-tenth of each were plated in 20 µL per well into Terasaki plates (Greiner HLA Terasaki 72 well plates, Millipore Sigma, Burlington, MA, USA). To facilitate the effector cell titration and the addition of target cells, the cell suspensions were first generated in a 96 well U bottom plate, from which 20 µL were transferred into each well of the Terasaki plate, plating in triplicate wells for each E:T ratio. For the Terasaki plates, no centrifugation steps were used before the 4 h incubation at 37 °C and 5% CO_2_. Following incubation, the Terasaki plate itself (without cell transfer) was analyzed by the S6 FlouroCore Analyzer using the Terasaki Plate Module of the TVA Suite.

### 2.6. The CFSE/PI TVA Assay

This variant of the TVA was performed as described above for the Calcein TVA, with the following steps differing: K562 cells were stained with the NK-CSFE dye (CTL), a ready to use CFSE-containing solution that has been optimized for imaging. The target cells were then washed, counted and plated, and effector cells added. At the end of the 4 h effector-target co-culture, 10 µL of NK-PI dye (CTL) was added to each well, a ready to use PI-containing solution that has been optimized for imaging. Imaging and counting were done on the ImmunoSpot^®^ S6 FluoroCore Reader, using the CFSE/PI module of its TVA software. Essentially, well images are captured with dedicated excitation and emission settings that permit to first selectively detect CFSE-stained cells (without cross-bleeding of PI staining), then PI-stained cells (without cross-bleeding of CFSE staining), followed by superimposition of cells in both categories to identify those cells that are double positive. In this module too, the corresponding graphs are automatically generated.

### 2.7. The Chromium Release Assay

The chromium release assay (CRA) was done following the standard protocol as described in detail in [17]. The CRA corresponds to the protocol described for the Calcein TVA above, except that the radioactive ^51^Cr salt, Na_2_^51^CrO_4_ (from Perkin Elmer, Waltham, MA), is used for labeling (target cells in 50 µL of RPMI-1640 with 10% FBS plus 50 µCi of Na_2_^51^CrO_4_ for one hour). At the end of the 4 h effector target cell co-culture in a 96 well U bottom plate, the plate is spun, and 100 µL of the supernatant collected, transferring it to polystyrene tubes for gamma counting on *a* WIZARD^®^ Gamma Counter (Perkin Elmer). Percent lysis is calculated according to the formula: Percent Specific Lysis *=* [(Experimental Release − Spontaneous Release)/(Maximum Release − Spontaneous Release)] × 100. The CRA has been performed at Pharmasan Labs., Inc. (Osceola, WI, USA), by D.R.R.

## 3. Results and Discussion

### 3.1. The Calcein-Based TVA 

#### 3.1.1. Live Target Cells Retain Calcein, Dead Target Cells Lose This Dye

**Figure 1:**
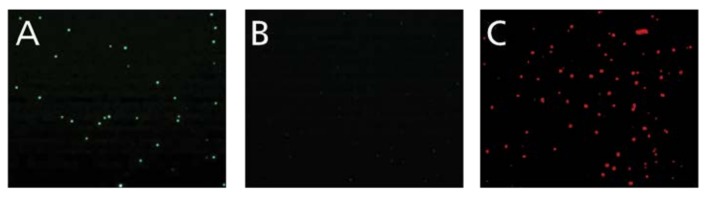
Calcein staining permits selective detection of live, but not of dead target cells. Live calcein-stained K562 cells (green) are shown in (**A**). The image has been acquired by an ImmunoSpot^®^ S6 FluoroCore Reader with the cells present in 100 µL culture medium in a 96 well flat bottom plate. (**B**) The same number of dead (ethanol-exposed) K562 cells has been plated with no calcein-stained cells detectable. (**C**) As in B, but PI was added to stain dead cells.

In our first effort for the development of a high-throughput and GLP suitable Target cell Visualization Assay (TVA), we utilized calcein-stained target cells. To establish the feasibility of the imaging approach, 5 × 10^3^ calcein-stained K562 cells were plated in 100 µL of cell culture medium into wells of a flat bottom 96 microtiter plate. Figure 1A shows that, in spite of the presence of the culture medium, individual stained cells can be readily imaged (and counted, see below). When the calcein-stained K562 cells were exposed to 95% ethanol, and thus killed, the dead cells no longer were calcein positive (Figure 1B), but became stained by PI (Figure 1C). Thus, only live K562 target cells retained calcein, dead target cells lost this staining. The data prove that the number of killed target cells can be calculated as the difference between the number of target cells present in the negative control wells, that do not contain effector cells, and in the experimental wells, that contain effector cells. Thus, the percentage of killing can be calculated for each E:T ratio. Based on this notion, we established the following formula for calculating % killing in the calcein assay: % Calcein Killing = (Average number of calcein-stained target cells counted in the triplicate experimental wells at the end of the assay/average number of calcein-stained target cells counted in the triplicate negative control wells) × 100.

**Figure 2:**
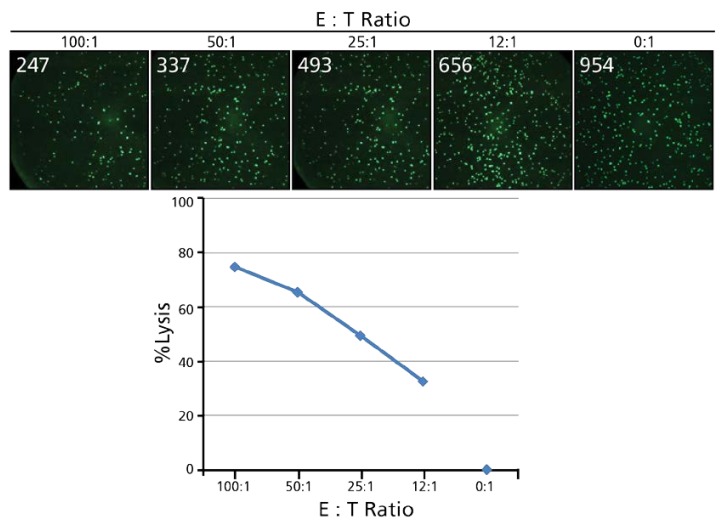
An example of the calcein-based TVA assay. A fixed number of calcein-labeled K562 target cells (5000/well) was incubated with effector cells (PBMC) at the specified effector: target (E:T) ratios in a round bottom 96 well plate for 4 hours. Subsequently, the cells were resuspended, and 3 × 50 µL from each assay well were transferred into triplicate wells of a flat bottom plate for imaging and counting in an ImmunoSpot^®^ S6 FlouroCore Reader with the NK-TVA™ Software (by CTL). The numbers of live cells counted per well are superimposed on the images. Negative control wells that contain target cells but no effector cells establish the zero killing control value. The difference in viable cell counts is expressed and plotted as % lysis as the function of the E:T ratio.

Based on the notion that dead target cells lose their calcein staining, we started to perform Calcein TVAs that were set up in an analogous fashion to traditional ^51^Cr release assays (CRA), incubating effector and target cells at various ratios, after which calcein-stained target cells were counted instead of ^51^Cr release measured. Figure 2 illustrates such an experiment. In this experiment, a decreasing number of effector cells (PBMC) are plated together with a constant number of calcein-labeled K562 target cells (4000 per well) resulting in effector: target (E:T) ratios ranging from 100:1 to 12.5:1. Effectors and targets are co-cultured for 4 h, after which 3 × 50 µL of the cell suspension present in each well of the original U bottom assay plate are transferred into a 96 well flat bottom plates for imaging and counting. Wells containing target cells with medium only, thus without effector cells, constitute the assay blank, or negative control. In the experiment shown in Figure 2, 954 cells were counted in the blank well: as ~4000 Calcein-labeled K562 cells were present in 200 µL in the original round bottom plate, of which one fourth were transferred 50 µL, theoretically 1000 labeled cells should be present in the imaged well. With 954 labeled cells actually counted, we can conclude that (a) essentially all cells K562 cells were labeled; (b) have been transferred; and (c) counted.

#### 3.1.2. In-House Validation of the Calcein TVA Using the ^51^Cr-Release Assay as the Compendial Method

**Figure 3:**
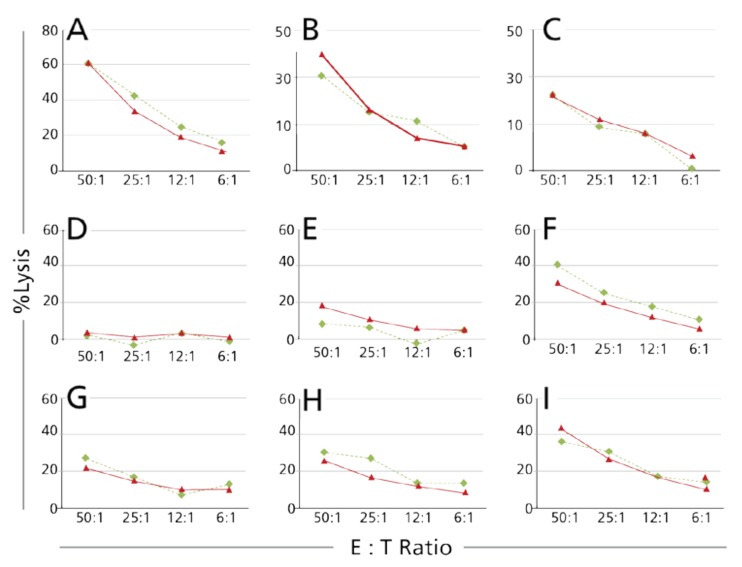
Comparing Calcein TVA with chromium release assay as the compendial method. The two assays were performed in parallel using the same PBMC, at the same effector to target (E:T) ratios and using the same numbers of K562 target cells. Each test condition was done in triplicate wells with the mean and standard deviation (SD) for these replicates shown. Red lines specify the results obtained with the chromium release assay, green lines those of the Calcein TVA assay. In Panels **A**–**I**, results are shown for individual donors, one in each panel.

Following general guidelines for cell-based assay validation, we started to explore whether the Calcein TVA is suitable for regulatory validation. As the CRA is still the gold standard for measuring cell-mediated cytolysis, we performed this assay in parallel with the Calcein TVA assay, whereby the same PBMC effector cells were tested simultaneously using both methods. PBMC from nine donors were tested in parallel for NK activity in this way, using K562 as targets. The results are shown in Figure 3. As can be seen, the two assays produced essentially overlapping killing curves for all 9 PBMC.

#### 3.1.3. In-House Establishment of the Intermediate Accuracy of the Calcein TVA

Intermediate Accuracy is defined as the reproducibility of test results when the same investigator performs the assay repeatedly. As all experiments communicated in this paper have been done with cryopreserved PBMC we could apply a reference sample strategy: by thawing of different aliquots of the same donor’s cells that have been frozen at the same time we were enabled to work with the same PBMC effector cells, that have the same, reproducible level of NK activity. PBMC from three donors were selected that showed high, intermediate, and low killing levels in previous screening experiments. On three subsequent days, one aliquot of each of these three PBMC was thawed, and tested by the same investigator (S.S). The results communicated in Figure S2 showed close to perfect reproducibility of the test results for all three PBMC donors and at all E:T ratios.

#### 3.1.4. Full, Independent, Third-Party Validation of the Calcein TVA

Based the above results that suggested that the Calcein TVA might be suitable for full regulatory validation, we sought out independent, third-party CLIA-certified laboratories that would be interested in adopting the TVA in lieu of the CRA, and would do a full validation of the TVA. By the time of submission of this manuscript, already two such laboratories have successfully concluded such validation and run the Calcein TVA routinely in a regulated environment (VN manuscript in preparation).

#### 3.1.5. Miniature Calcein TVA Run in Terasaki Plates

**Figure 4:**
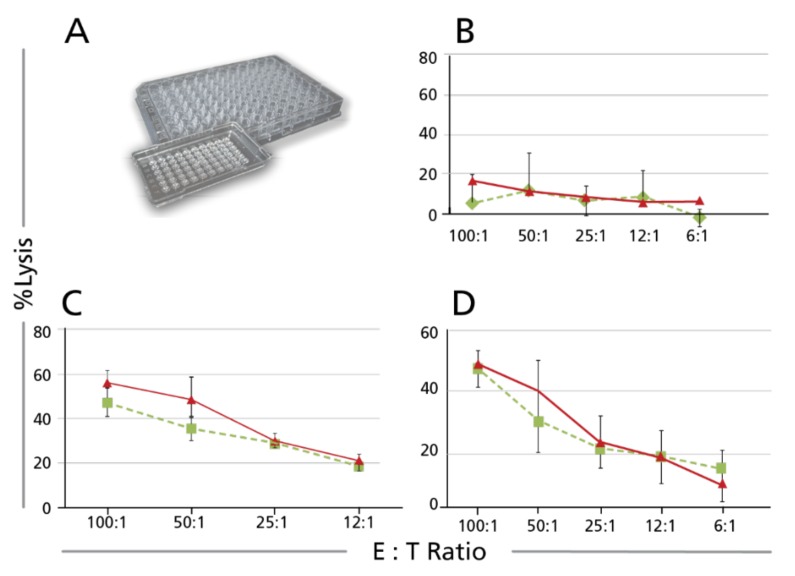
The Mini Calcein TVA assay compared with that performed in 96 well format. (**A**) shows a photograph of a Terasaki plate next to a regular 96 well plate. Calcein TVA Assays were done in parallel in both plate formats, using the same effector-target cell mixtures, of which 200 µL were plated into the 96 well U bottom plates (green lines), and 20 µL per well into the Terasaki plate (red lines). The results for three donors tested in parallel are shown in panels (**B**–**D**), respectively.

Calcein-TVA assays performed in 96 well plates require exactly as many effector cells as the corresponding CRA. PBMC of patients are, however, many times limited, and this particularly holds true for samples obtained from children, elderly or terminally ill patients. It also applies for testing non-PBMC primary effector cells, such as bronchial alveolar lavage cells. To account for such scenarios when limitations in effector cell numbers would preclude performing a cell-mediated cytotoxicity assay, we made an effort to miniaturize the TVA. In the Mini TVA variant, instead of plating the effector and target cells in 200 µL per well into a 96 well plate, one-tenth of each cell type is plated into wells of a Terasaki plate, in 20 µL per well. In this way, the assay can be performed with one-tenth of effector (and target) cells needed. Figure 4 shows that the two assay formats provide comparable results. In addition to conserving cell material, a major advantage of the Mini Calcein TVA assay is that the target cells can be imaged and counted directly in the Terasaki plate, without the need of cell transfers into another plate, as is the case with the 96 well format. Direct imaging and counting in the Terasaki format also enable kinetic measurements of killing: the plate can be scanned repeatedly at intervals measuring how killing progresses.

### 3.2. The CFSE/PI TVA

The Calcein TVA permits counting target cells that stay alive after cytotoxicity testing—the target cells that have been killed “disappear” as they lose their label. It should be of advantage, however, if one could count simultaneously not only the live target cells, but also those that have been killed. First, one gains an independent internal confirmation of killing when it is observed that while live target cell numbers decrease the number of dead target cells increase; false negative/positive data due to errors in pipetting, cell transfers, or counting can be excluded when both criteria for killing are established. Second, one might expect the detection of dead target cells to be a more sensitive and quantitative measure of killing than the decrease in numbers of live target cells. Thus, for example, in order to measure 10% killing by the decrease of numbers of live target cells, 500 target cells need to be killed per well of 5000 plated. In contrast, if the detection of killed target cells operates at single cell resolution, in theory, even the killing of a single target cell would be detectable, and the killing of a dozen target cells, occurring in replicate wells, might already constitute highly significant evidence for ongoing cytolytic activity.

**Figure 5:**
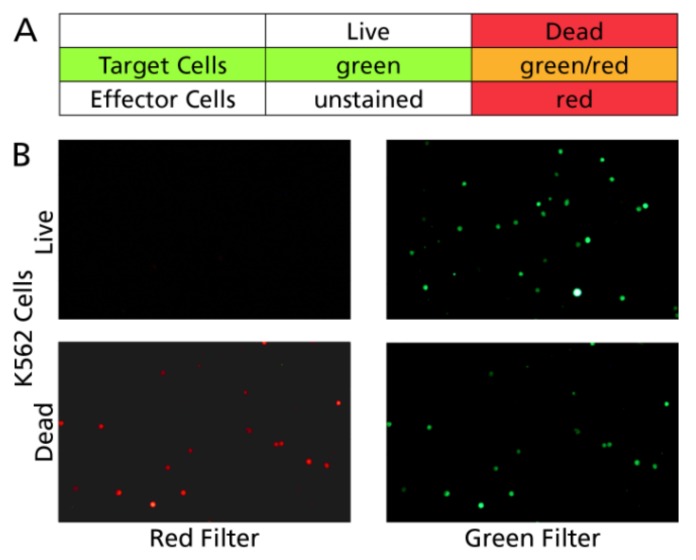
Principle of discriminating live and dead target cells by CFSE/PI staining. (**A**) While dead target cells retain the CFSE stain, and PI stains dead target and dead effector cells alike, only the dead target cells will become double positive. (**B**) Live target cells, when imaged, are visible in the green channel only, without cross-bleeding into the red channel. Dead target cells are positive in both, and by superimposition of the images can be automatically identified by the TVA software as double positive.

To detect living and killed target cells, we used the CFSE/PI staining approach, the rationale of which is illustrated in Figure 5A. The target cells are stained with CFSE prior to the assay. Unlike calcein, however, killed target cells are expected to stay CFSE stained due to the covalent binding of the dye to cytoskeletal proteins. By counterstaining the cell culture at the end of the killer assay with PI, these killed target cells become double positive (CFSE and PI positive). In contrast, dead effector cells will be singly PI positive. Figure 5B shows that, indeed, the two colors do not cross-bleed: live cells light up only in the green channel, and dead cells in both.

Detecting double positive, i.e., killed target cells, among primary effector cells represents a challenge, however. Let us consider a well in which 5 × 10^5^ PBMC are plated along with 5 × 10^3^ target cells, corresponding to the typical 100:1 E:T ratio in a 96 well plate. If the assay is done with thawed cryopreserved PBMC, or PBMC that have been stored/shipped, one might expect 10% (or even more) of these PBMC to be dead. In this example, with 5 × 10^5^ PBMC plated of which 10% are dead, there would be 5 × 10^4^ dead PBMC present in the well (along with 4.5 × 10^5^ live PBMC). If 10% of the target cells are killed during the assay, there will be 500 killed target cells present in this well. In this example, dead PBMC (single-positive for PI/red) would outnumber 100:1 killed target cells (which are double positive)! We, therefore, tested experimentally whether precise counting of these few killed targets is possible in the presence of an excess of dead effector cells.

#### 3.2.1. Unambiguous Detection of Dead Target Cells among an Excess of Dead Effector Cells

**Figure 6:**
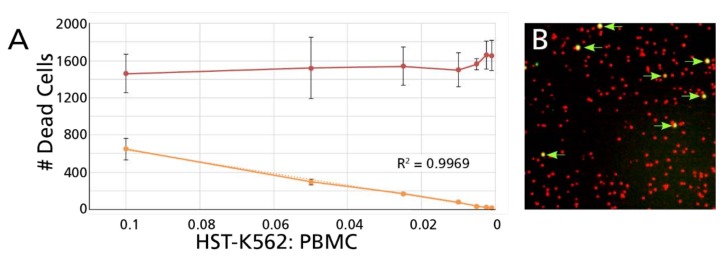
Detection of individual dead K562 target cells within an excess of live and dead PBMC effector cells. (**A**) Fixed number of 12,500 thawed cryopreserved PBMC were plated into a flat bottom 96 well plate whereby 12% of these PBMC were dead as measured by acridine-orange/PI cell viability counting. To these PBMC, a decreasing number of heat-shock-treated (HST) K562 cells were added—these K562 cells were stained with CFSE prior to the HST. Forty-seven % of these HST treated 562 cells proved to be dead by acridine-orange/PI cell viability counting. The highest number of HST-K562 cells was 1250 cells per well, corresponding to a 1:10 (0.1%) HST-K562 to PBMC ratio. For each subsequent test case, the numbers of HST K562 cells was decreased by half while keeping the PBMC numbers constant. Each serial dilution was plated in triplicate wells. In the next step PI was added, and the cells were counted by the ImmunoSpot^®^ S6 FloroCore Reader using the TVA platform. The mean spot counts for single positive red PBMC (PI-only stained) are represented by the red symbols with SD shown for the triplicate wells. CFSE/PI double positive dead target cells were also automatically counted with the mean counts and SD represented by the orange symbols. (**B**) A magnified well segment is shown with the double positive cells highlighted with an arrow.

We used a freeze-thawed PBMC sample in which 88% of the cells were found to be viable and 12% dead after thawing. These cells were plated at a constant number of 12,500 PBMC per well, with a serial dilution of heat shock-treated (HST) K562 cells that had been CFSE-stained prior to the HST. (Due to the robustness of these tumor cells, HST killed only half of the K562 cells). PI was added to each well, followed by imaging and counting of stained cells. The number of double positive (CFSE/PI positive “killed target”) cells decreased with close to perfect linearity (*R*^2^ = 0.9969) while the number of single positive red (PI, dead PBMC) stayed constant while the expected and counted cell numbers in each condition closely matched up (Figure 6). Thus, even a single killed K562 target cell could be detected within the 12,500 PBMC, of which 1500 were dead PBMC.

#### 3.2.2. The Calcein TVA and the CFSE/PI TVA Have Similar Sensitivity Detecting Killing Based on Counts of Live Target Cells 

**Figure 7:**
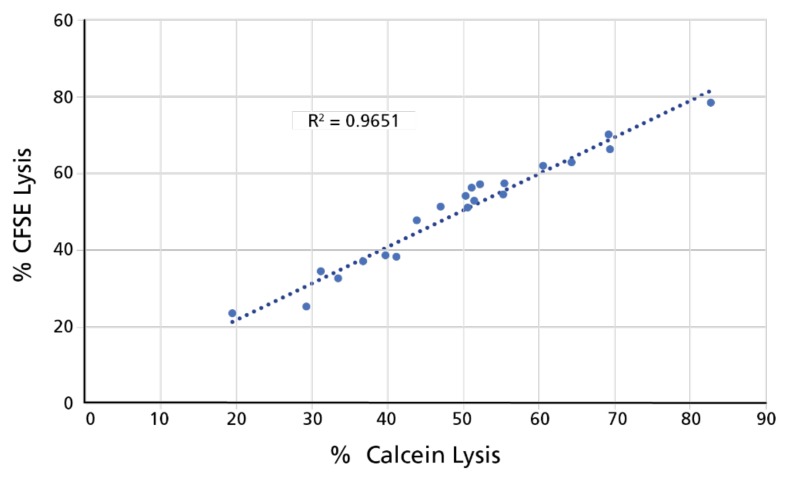
Results of % killing established by Calcein TVA and CFSE/PI TVA closely match. PBMC of 21 donors were tested in the two assays side by side. The results obtained for the 100:1 effector to target ratio are plotted here against each other. The correlation quotient for the closeness of fit is shown.

In Calcein TVA, the counting of live target cells can be done directly, as dead targets lose the dye. The counting of live target cells in the CFSE/PI assay, however, is dependent on the correct recognition of not only the single CFSE-positive (live target cells) but also of the CFSE/PI double positive (killed target) cell fractions. The live target cell number in the CFSE/PI assay, therefore, is calculated by the numbers of all CFSE-positive cells (all target cells, live or dead) minus the dead target cell numbers (the CFSE/PI double-positive number). Once the number of live targets has been established in this way, percentage killing is calculated in the same way as in the Calcein TVA assay [% killing = (live cells counted/live cells in negative control) × 100]. We performed Calcein TVAs and CFSE/PI TVAs in parallel, testing the same PBMC for NK activity towards K562 target cells (that were either labeled with calcein or with CFSE prior to testing). The resulting curves were superimposable—in Figure 7 results are shown for the 100:1 data point for 21 PBMC for which the comparison of the two assay formats was done. The correlation was close to perfect, with *R*^2^ = 0.9651.

#### 3.2.3. Counts of Dead Target Cells in the CFSE/PI TVA Confirm Results Established by Decreased Numbers of Live Target Cells 

**Figure 8:**
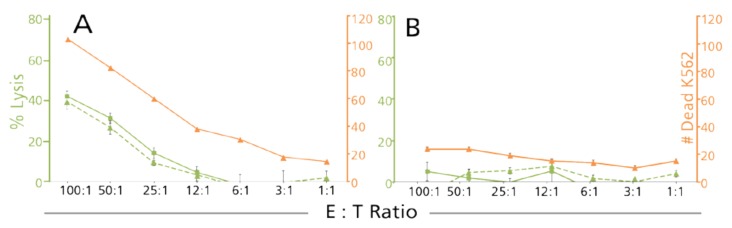
Concordant detection of killing by counting dead- and % reduction in numbers of live target cells. Sixty-four PBMC samples were tested in the Calcein TVA (green dashed lines referring to the green Y-axis on the **left**), and the CFSE/PI TVA in parallel, whereby in the latter the dead target cell counts are represented by the orange lines (referring to the orange Y-axis on the **right**), and the % reduction in live target cells (% CFSE lysis) by the solid green lines (referring to the green Y-axis on the **left**). Representative results are shown for a PBMC donor whose cells did exert NK killing (**A**), and for a donor whose PBMC lacked cytolytic NK activity (**B**).

CFSE-PI TVAs and Calcein TVAs were done on additional donors (total *n* = 64) comparing the dead target cell counts with the decrease of live target counts as established in both assay formats. The results showed that in all cases when the numbers of target cells dropped by more than 15% (thus killing became evident by the criterion of target cell decline) killing also become evident by the presence of increased numbers of dead target cells (55 of 64 donors). Results for a representative donor in this category are shown in Figure 8A. In 14% of the test cases (9 of 64 donors) no significant killing was seen with either of the three approaches (Figure 8B). The counts of dead target cells, therefore, provide an independent internal control for the presence, or absence, of killing.

In theory, an ideally counted CFSE/PI assay should reveal every single killed target cell. For example, if live cell counting (by the CFSE/PI and/or the calcein method) reveals that 30% of 1,250 target cells plated per well have been killed (that is, 875 live cells can still be detected), in that case one would expect to count 375 dead target cells in that well. For 28 test cases we compared the expected and counted the number of dead target cells: in 17 test cases (61%) the expected and detected dead target cell numbers closely matched up. In the remainder of the 11 test cases, either slightly more (6 of 28) or slightly less (5 of 28) dead target cells were detectable than suggested from % killing established by the decrease of live target cells. The higher number of dead target cells might reflect on the theoretically expected higher sensitivity of the single cell measurement. The likely explanation for occasionally detecting fewer than expected lysed target cells could be that such cells either disintegrated or became phagocytosed in the cell culture.

In all above assays, PBMC were used as effector cells to determine their cytolytic activity on K562 target cells. Based on the established susceptibility of K562 cells to killing by NK cells, it was assumed that NK cells within the PBMC population are the actual effector cells. To verify this notion, we tested unseparated, and NK-depleted PBMC in parallel. While the unseparated PBMC exerted cytolytic activity on K562 cells (Figure S3A), no killing was seen with the NK-depleted PBMC (Supplementary Figure S3C). NK depletion, as expected, did not affect the magnitude of the CD8 T cell recall response to CEF peptides (Supplementary Figure S3B vs. S3D).

## 4. Conclusions

The data presented here establish the feasibility of single-cell imaging-based high throughput cytotoxicity testing. This TVA approach does not rely on radioactivity, yet was shown to have the same sensitivity as standard ^51^Cr-release assays. Unlike the CRA, however, TVA is directly quantitative by relying on the counting of individual target cells. Also unlike with the CRA, TVA generates tamper-proof audit trails that make the assay ideally suitable for work in a regulated environment. The robustness of the TVA assay established here further facilitates its adaptation to such an environment. The CRA establishes only one parameter for cytotoxicity: the percentage of target cells killed. The Calcein TVA assay—introduced here—does the same, with similar sensitivity. Unlike the CRA (or flow cytometry-based live/dead target cell counting) the Calcein TVA can be miniaturized to require only one-tenth the numbers of effector cells—a very important modification when the numbers of effector cells are limiting.

The CFSE/PI modification of the TVA allows one to assess not only % killing based on the reduction of numbers of live target cells—and doing so with the same sensitivity as the CRA or Calcein TVA, but additionally also detects the numbers of dead target cells in the same wells. Thus, an internal control for killing is established using two simultaneous yet independent read-outs for killing. Additionally, CRA (and flow cytometry-based target cell monitoring) are confined to measuring killing at a single time point, typically after 4 h of effector-target cell co-culture. TVA, in contrast, allows one to image wells serially, enabling measurements of the kinetics of cytolysis. Finally, due to the spontaneous leakage of ^51^Cr from the labeled target cells, ^51^Cr release assays are confined to relatively short periods of effector: target cell exposures. As dyes like CFSE are retained within labeled target cells for several days, the assay duration can be extended allowing effector cells to live up to their nature as “serial killers”: in this way, low frequency and weak cytolytic activity can be amplified, becoming detectable. The CFSE/PI TVA involves dual color cell counting. We presently are working on extending TVA for multi-color analysis, staining different target cells with dyes of non-overlapping spectra, thus allowing one to test simultaneously the lysis of several target cells within the same well, utilizing a single effector cell population, and to test negative control cell lines along with those that are expected to be killed.

## Supplementary Materials

The following are available online at http://www.mdpi.com/2073-4409/7/5/35/s1, Figures S1–S3.
