# Supplementary material for: High-Throughput GLP-Capable Target Cell Visualization Assay for Measuring Cell-Mediated Cytotoxicity
Source: Cells. 2018 Apr 24;7(5):35. doi: 10.3390/cells7050035 (PMC5981259; doi:10.3390/cells7050035)
Supplement: Supplementary file 1 [file cells-07-00035-s001.pdf]

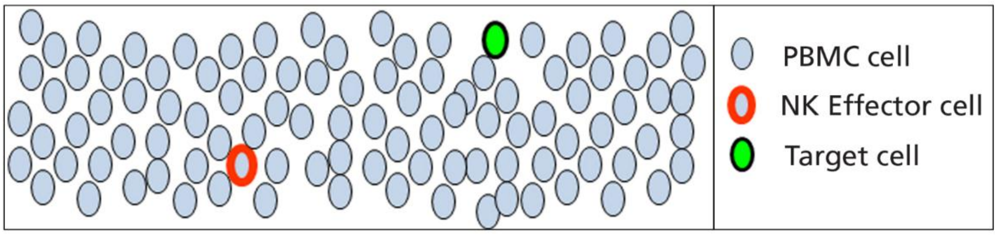

Figure S1. The odds of an effector cell meeting a target cell. When PBMC are used as "effector cells", the highest effector to target cell ratio (E:T) is typically 100:1. In this figure, 100 PBMC are depicted. Of these only few cells are indeed effector cells – in this illustration 1 in 100, highlighted in red. At 100:1 E:T ratio, one target cell would be present, shown here in green. The figure illustrates that even when effector cells are present in relatively high frequency (like with NK cells), and even at the highest effector target ratio (100:1), the odds for effector cells meeting the target cell are suboptimal. At lower effector: target ratios, and when effector cells occur at substantially lower frequencies, as with most antigen-specific T cells, these odds are even worse. Ideal assays for measuring cytotoxicity, therefore, can detect even few individual target cells that have been killed, rather than relying on a reduction in numbers of all live effector cells present in a well.

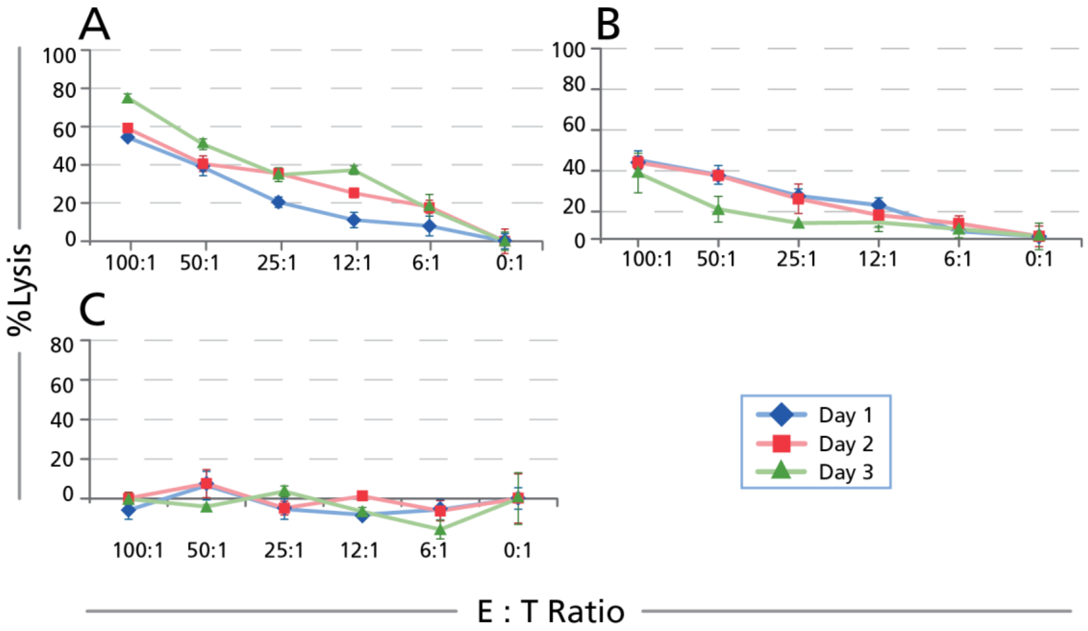

Figure S2. Intermediate accuracy of the Calcein-TVA. (A) PBMC from a donor who showed high NK activity in previous screening experiments, a donor who showed intermediate (B), and a third donor displaying no cytolytic activity (C) were tested. Aliquots of the same PBMC reference sample batch were thawed on three subsequent days and tested for cytolytic NK activity against K562 target cells using the Calcein TVA assay.

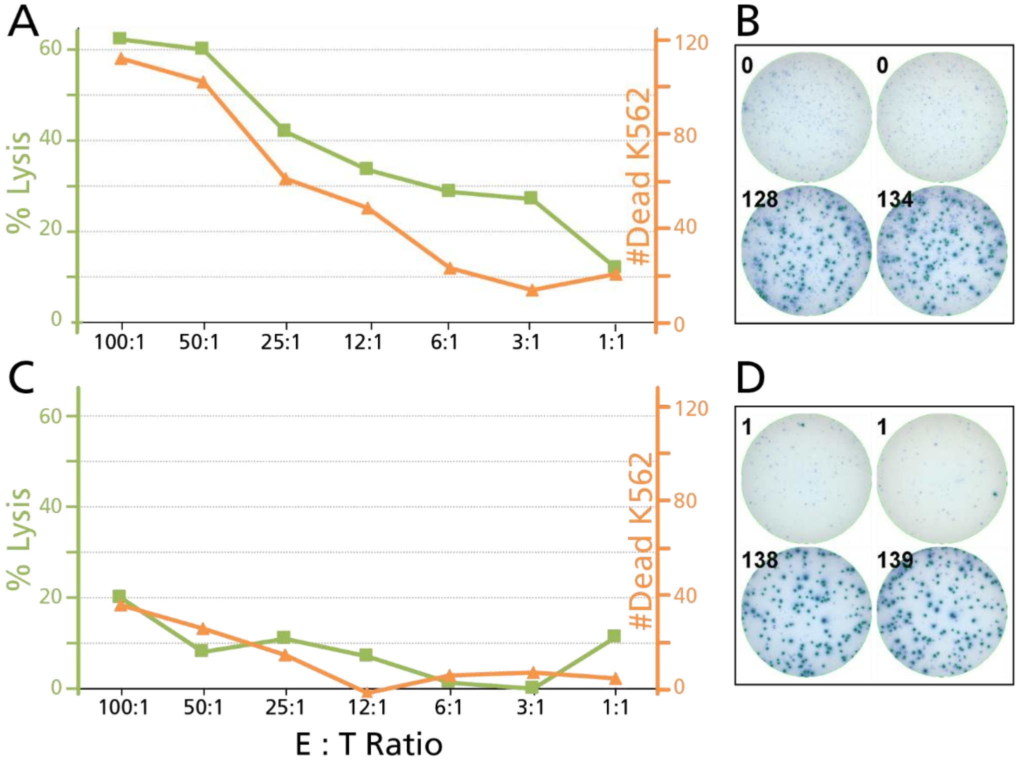

Figure S3. Cytolysis of K562 target cells is mediated by the NK subpopulation of PBMC. A PBMC donor was selected whose cells showed strong cytolytic activity towards K562 target cells, and these PBMC were tested in a CFSE/PI TVA at different effector to target ratios. For these unseparated PBMC, the % reduction of live cells (% lysis, the green line, referring to the green Y-axis on the left), and the numbers of dead K562 cells (orange line, referring to the orange Y-axis on the right) are shown in (A). In parallel, the same PBMC were tested after NK cell-depletion using magnetic beads (C). As controls, IFN-γ ELISPOT assays were done to test the frequency of CEF-peptide pool-reactive CD8 cells in the unseparated PBMC (B) vs. the NK depleted PBMC (D). In each of these ELISPOT panels, duplicate wells are shown on the top for spontaneous cytokine production, and duplicate wells underneath showing the assay wells containing the CEF antigen. The ELISPOT counts are shown in the upper left corner of each well image.
